# Lehrfilm über das geriatrische Basisassessment in der hausärztlichen Versorgung – Methoden filmischer Authentizität

**DOI:** 10.1007/s00391-021-01993-6

**Published:** 2021-11-12

**Authors:** G. Stiller, R. Stegemann, K. Afshar, M. Marschollek, M. Behrends

**Affiliations:** 1 Peter L. Reichertz Institut für Medizinische Informatik, TU Braunschweig und Medizinische Hochschule Hannover, Carl-Neuberg-Str. 1, 30625 Hannover, Deutschland; 2 Institut für Allgemeinmedizin und Palliativmedizin, Medizinische Hochschule Hannover, Hannover, Deutschland

**Keywords:** Allgemeinmedizin, Medizinische Ausbildungsforschung, Lehrfilmproduktion, Filmrezeption, Medizinstudium, Family medicine, Medical education research, Educational film, Film perception, Medical education

## Abstract

**Hintergrund:**

Die Bedeutung der Versorgung älterer Personen in der hausärztlichen Praxis erfordert die Vermittlung von Handlungs- und Begründungswissen zur Durchführung eines geriatrischen Basisassessments (GBa) bereits im Medizinstudium. Handlungsbedarf zeigt sich dabei insbesondere im Hinblick auf eine realistische Wahrnehmung altersbedingter Einschränkungen durch Studierende. Der Beitrag beschreibt anhand eines Filmprojekts an der Medizinischen Hochschule Hannover (MHH), wie die hausärztliche Versorgungssituation für Studierende authentisch vermittelt werden kann.

**Methodik:**

Verschiedene filmische Mittel zur Herstellung von Authentizität wurden angewendet. Im Lehrmodul Allgemeinmedizin an der MHH wurde der Film eingesetzt. Die studentische Evaluation untersuchte die emotionale Wirkung des Filmgeschehens auf die Studierenden, und ob die Darstellung des GBa als authentisch wahrgenommen wird.

**Ergebnisse:**

Die Authentizität der filmischen Darstellung hat die Mehrheit der Studierenden emotional berührt. Die Vermittlung der Komplexität der Behandlung älterer Menschen und die Darstellung der besonderen hausärztlichen Funktion in der geriatrischen Versorgung sind durch den Lehrfilm gut gelungen.

**Diskussion:**

Die Studierenden erkannten die Notwendigkeit des GBa und fanden die Darstellung überwiegend realistisch. Individuelle filmische Rezeptionsweisen und Vorerfahrungen beeinflussen auch die Wahrnehmung der filmischen Gestaltungsform im Hinblick auf Realitätsdarstellung.

## Abstract (en)

**Background:**

The relevance of care of older persons in general practice requires the conveyance of procedural and conditional knowledge of the geriatric basic assessment (GBA) even during medical studies. There is a need for action with respect to student knowledge on specific problems of older patients. This paper describes how the primary care situation can be made tangible for students based on a film project at the Hannover Medical School (MHH).

**Method:**

During film production, strategies for creating authenticity were applied using cinematic means. The film is used in the teaching module of family medicine at MHH. Student evaluation investigated whether the educational film provided an emotional experience and whether the presentation mode of the GBA was perceived as authentic.

**Results:**

The majority of students were emotionally touched by the film. The educational film was successful in conveying the complexity of care of older people and in presenting the special role of family physicians in geriatric care.

**Discussion:**

The students recognized the necessity of the GBA and found the representation predominantly realistic; however, individual cinematic reception and previous experiences also influence the perception of the cinematic form with respect to the representation of reality. This method might improve the conveyance of authenticity in educational videos in medicine.

## Einleitung

Die Versorgung älterer und damit nicht selten auch multimorbider Menschen ist eine wesentliche Aufgabe in der hausärztlichen Versorgung. Dabei ist es wichtig, altersbedingte Einschränkungen zu erkennen und Unterstützungsangebote für die Bewältigung von Alltagsproblemen anzuregen. Mit dem geriatrischen Basisassessment (GBa), das seit 2005 Teil des Leistungskataloges der hausärztlichen Versorgung ist, liegt ein geeignetes Testverfahren vor, welches den gesundheitlichen Zustand älterer Patient*innen ganzheitlich erfasst und auf Indikationen für eine weitergehende Diagnostik und Therapie hinweist [6, 18].

Aufgrund der Relevanz für die hausärztliche Versorgung entstanden Bestrebungen, die erforderlichen Kompetenzen zur Durchführung eines geriatrischen Assessments in der medizinischen Ausbildung curricular zu verankern [4, 21] und geeignete Lernziele zu formulieren [10, 17]. In der aktuellen Fassung des Nationalen Kompetenzbasierten Lernzielkatalog Medizin (NKLM 2.0) [12] wird das geriatrisches Assessment dementsprechend unter den Konsultationsanlässen und den klinisch-praktischen Fertigkeiten aufgeführt. Auch im Hinblick auf die studentische Wahrnehmung des Alters zeigt sich, dass Studierende unzureichende Vorstellungen von den Lebensumständen sowie den spezifischen Problemlagen und Krankheitsbildern älterer Menschen haben [22]. Mittels spezifischer Lehr- und Lernszenarien sollte eine realistische Perspektive auf das Alter vermittelt werden [14, 20].

An der Medizinischen Hochschule Hannover (MHH) ist das GBa als Teil des Unterrichtsmoduls zum Hausbesuchs [11] seit dem Studienjahr 2013/2014 Bestandteil der allgemeinmedizinischen Lehre. Die Lernziele zum Kurs „Hausbesuch und geriatrisches Basisassessment“ im Unterrichtsmodul der Allgemeinmedizin umfassen das Wissen um die spezifischen Aufgaben der hausärztlichen Versorgung im geriatrischen Bereich, die Kenntnis der relevanten Assessmentverfahren und das Reflektieren dieser aus Patienten- und Angehörigensicht.

Um eine stärkere Patientenorientierung zu ermöglichen, wurde im Sommer 2016 ein Lehrfilm erstellt, der das GBa im Rahmen eines Hausbesuches zeigt und die Kommunikation und Interaktion mit den älteren Patient*innen im häuslichen Umfeld darstellt. Dabei wurde ein filmisches Darstellungskonzept umgesetzt, das verschiedene filmische Mittel nutzt, um Realitätsnähe und Authentizität zu erreichen.

Eng verbunden mit einer als realitätsnah empfundenen filmischen Darstellung ist der Begriff der Authentizität. Der im Diskurs des Dokumentarfilms wichtige Begriff der „Authentizität“ bezieht sich entsprechend dem griechischen Ursprungswort „authéntes“ auf die eigenhändige Fassung von Schriftstücken und steht in heutiger Sicht für Zuschreibungen der Echtheit, Zuverlässigkeit, Eigenhändigkeit und Glaubwürdigkeit [9]. Die etymologische Entwicklung des Begriffs „Authentizität“ beinhaltet insofern schon vom Wortursprung her nicht die Wiedergabe von Realitätsausschnitten, sondern legt das Hauptgewicht auf die eigenhändige Produktion von Werken. Authentizität wird durch den Darstellungsprozess geschaffen. Dabei ist es entscheidend, dass das im Film dargestellte Ereignis auf die Betrachter*innen glaubwürdig wirkt. Im Prozess der Wahrnehmung entsteht Authentizität durch die Annahme von Glaubwürdigkeit [5].

Im Film kann eine authentische Wirkung durch verschiedene Gestaltungselemente befördert werden:

- Reale Protagonisten, die in Mundart sprechen oder eine Fachsprache verwenden, wirken lebensnah.
- Der Dreh an realen Orten mit Originallicht kann zwar zu Einbußen der Abbildqualität führen, erhöht aber die Glaubwürdigkeit des Dargestellten, auch wenn es dadurch zu Einbußen der Abbildqualität kommen kann.
- Einstellungsgrößen der Kamera erzeugen den Eindruck von Nähe oder Distanz.
- Techniken der Montage beeinflussen das zeitliche Erleben des Filmgeschehens und strukturieren die Wahrnehmung der filmischen Handlung. Die Schnittfrequenz des Films bestimmt das Tempo der Erzählung. Auslassungen mit vielen Schnitten erzeugen Dynamik und Schnelligkeit; eine Montageform mit wenigen Schnitten führt zu einer ruhigen Erzählweise mit Spielraum für Beobachtungen.
- Durch Schnitt und Gegenschnitt mit jeweils einer sichtbaren Person kann der Eindruck eines Dialogs erzeugt werden.
- Durch Texteinblendungen und andere Mittel kann die Geschlossenheit der filmischen Realität aufgebrochen werden.
- Indem der Erstellungsprozess des Films offenkundig gemacht wird, entsteht auf der Metaebene eine Selbstreflexivität. Die Sichtbarmachung des Produktionsprozesses belegt dabei die Echtheit des Gezeigten.

Dieser Beitrag untersucht, ob mit den gewählten filmischen Mitteln ein authentischer Eindruck hinsichtlich des GBa in der häuslichen Versorgung erreicht werden konnte, ob eine emotionale Wirkung entfaltet und ob mit der gewählten Darstellungsform die patientenorientierte Wissensvermittlung in Bezug auf geriatrische allgemeinmedizinische Kompetenzen gelingt.

## Methoden

### Lehrfilm zum geriatrischen Basisassessment

Der Lehrfilm *Der Hausbesuch in der Allgemeinmedizin und das geriatrische Basisassessment* hat eine Länge von 23,30 min (exkl. Vor- und Abspann). Darsteller sind das Ehepaar K. und ihr Hausarzt. Gedreht wurde der Film am und im Einfamilienhaus des Ehepaars K, das in einer Kleinstadt gelegen ist. Der Film zeigt den Hausbesuch eines niedergelassenen Hausarztes mit seiner Ankunft am Wohnhaus der Patient*innen, der Durchführung eines geriatrischen Assessment mittels Manageable Geriatric Assessment (MAGIC), dem Test zur Demenz-Detektion (DemTect) und der abschließenden Wohnungsbegehung. Es gab ein handlungsorientiertes Drehbuch, allerdings ohne festgelegte Vorgabe der Dialoge, damit die Darsteller*innen frei sprechen konnten.

**Figure 1:**
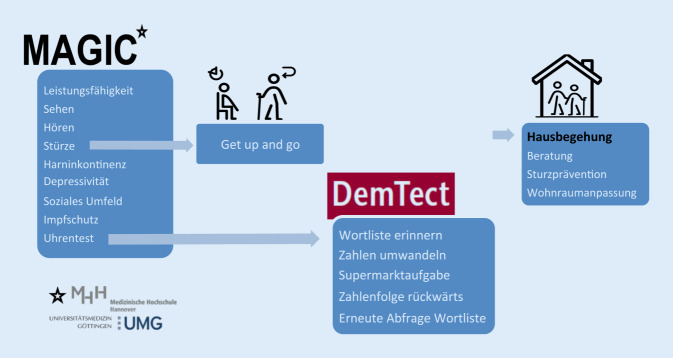

Das GBa erfolgt im Film auf Grundlage des MAGIC [1], das entsprechend der Leitlinie „Geriatrisches Assessment in der Hausarztpraxis“ der Deutschen Gesellschaft für Allgemeinmedizin und Familienmedizin (DEGAM) [2] geeignet ist, altersbedingte Einschränkungen zu erfassen, die im medizinischen Routinebetrieb oftmals nicht erkannt werden (Abb. 1). Um den Bedarf eines Assessments abzuklären, empfiehlt die DEGAM-Leitlinie, ältere Patient*innen mittels zweier Signalfragen (Fühlen Sie sich voller Energie? Haben Sie Schwierigkeiten, eine Strecke von 400 m zu gehen?) anlasslos oder auch regelmäßig nach deren Konstitution zu befragen: Auf Basis dieser Vorbefragung erfolgte die Auswahl von Herrn K. als Hauptprotagnisten im Film.

Der Lehrfilm beginnt mit der Ankunft des Hausarztes vor dem Haus des Ehepaares K., um einen Eindruck vom sozialen Wohnumfeld zu vermitteln. Geöffnet wird die Tür von Frau K., die den Hausarzt bittet, im Wohnzimmer Platz zu nehmen, wo bereits ihr Ehemann wartet. Nach der Begrüßung erläutert der Hausarzt in einfachen Worten, welche Untersuchungen während des Hausbesuches geplant sind. Die Ehefrau ist die agilere von beiden und kommentiert den Gesundheitszustand ihres Mannes. Diese Anfangssituation im Wohnzimmer macht das Verhältnis des Ehepaares deutlich. Mit der an den Patienten gerichteten Aufforderung, gemeinsam zum Esstisch zu wechseln, stehen beide auf. Aufgrund erster erkennbarer Mobilitäteinschränkungen benutzt Herr K. einen Gehwagen.

Am Esstisch angekommen, setzen sich Hausarzt und Herr K. über Eck gegenüber, wodurch der Betrachter die Personen nahezu im Profil sieht. Die Fragen des Arztes und die Antworten und Reaktionen des Patienten sind durchgängig in einer halbnahen Einstellung gefilmt und nicht mit Schnitt und Gegenschnitt im Sinne einer szenischen Auflösung montiert. Beide Protagonisten sind vielmehr gleichzeitig im Bild und können mit ihrem jeweiligen Verhalten, ihren Gesten und ihrer Mimik wahrgenommen werden.

Der Hausarzt beginnt das Gespräch mit den im MAGIC formulierten Fragen zur Bestimmung der Alltagseinschränkungen. Zur Testung der kognitiven Leistung wird dann der Uhrentest [15] durchgeführt. Dieser stellt die Schlüsselszene des Films dar, da hier erstmalig das Ausmaß der kognitiven Belastung von Herrn K. deutlich wird. Der Test wird im Film in voller Länge gezeigt, wodurch das Ringen von Herrn K. um die Lösung der Aufgabe wie auch der Grad seines Scheiterns für den Betrachter deutlich werden. Nach dem Uhrentest folgt der ‚Timed-up-and-go‘-Test [2] zur Überprüfung der Beweglichkeit und der Erkennung der Sturzgefährdung. Auch hier zeigen sich Einschränkungen. Den anschließenden Demenztest (DemTect) [7, 8] bricht Herr K. dann entmutigt ab, da er die erforderlichen Aufgaben nicht bewältigen kann.

Bei der anschließenden Besprechung thematisiert der Hausarzt sowohl die eingeschränkte motorische Leistungsfähigkeit als auch die kognitiven Probleme. Um dem entgegenzuwirken, verordnet der Arzt Herrn K. Ergotherapie. Durch Einblendungen des Auswertungsergebnisses des DemTect wird den Studierenden der Verdacht einer demenziellen Erkrankung aufgezeigt.

Abschließend führt der Hausarzt gemeinsam mit der Ehefrau des Patienten eine Begehung der Wohnung durch, um Gefahrenstellen für ein Sturzrisiko zu identifizieren. Auf die Empfehlung, einen Teppich als potenzielle Stolperfalle zu entfernen, reagiert die Ehefrau allerdings ablehnend.

Filmisch wird der Hausbesuch als unkommentierte Beobachtung dargestellt. Einblendungen der handschriftlichen Eintragungen des Patienten Herr K. im Assessmentbogen ergänzen die filmische Erzählung. Es gibt keine vorangestellten Informationen zum gesundheitlichen Zustand des Patienten. Im Schnitt wurde die Dauer der Untersuchung ungekürzt beibehalten.

### Untersuchung der Filmwirkung

An der MHH wurde der Lehrfilm erstmals im Studienjahr 2016/2017 im Modul Allgemeinmedizin eingesetzt, das im 3. Studienjahr verortet ist.

Der Film wurde im Hörsaal allen Studierenden gezeigt und diente anschließend als Basis für den Kleingruppenunterricht zur Bearbeitung von Aspekten des GBa. Zu der regulären Modulevaluation erfolgte eine ergänzende Befragung der Studierenden zu dem eingesetzten Lehrfilm. Die Evaluation erfolgte papierbasiert anhand eines teilstandardisierten Fragebogens nach Unterrichtsende. Der Fragebogen umfasste 9 Items, davon 7 geschlossene und 2 offene Fragen. Bei den geschlossenen Fragen sollten Aussagen anhand einer 6‑stufigen Likert-Skala von 1 „Trifft voll zu“ bis 6 „Trifft überhaupt nicht zu“ bewertet werden. Drei Items bezogen sich auf die Einschätzung der Komplexität der Behandlung, der generellen Notwendigkeit der verschiedenen Assessments und der Darstellung der hausärztlichen Koordinationsfunktion. Die im Lehrfilm angewendete filmische Darstellungsform wurde anhand einer Frage zur Realitätsnähe der gezeigten Situation bewertet. Die emotionale Wirkung des Films wurde anhand der Beurteilung einer der Schlüsselszenen des Films untersucht, indem die Studierenden gefragt wurden, inwiefern sie das Zuschauen bei der Durchführung des Uhrentests emotional berührt hat, sowie durch die Frage, inwiefern das Ergebnis des Assessments dem Ersteindruck entsprach. Die Gesamtbewertung des Films erfolgte anhand einer 6‑stufigen Skala entsprechend dem Schulnotensystem. Die offen formulierten Fragen „Welche Aspekte des Lehrfilms bewerten Sie positiv“ bzw. „… bewerten Sie negativ“ erlaubten die Eingabe von Freitextantworten. Befragt wurden die Studierenden auch, ob sie bereits Kontakt zu älteren Patienten hatten. Weitere Daten zur Charakteristik der Studierenden wurden zur Wahrung der Anonymität nicht erhoben.

Die Befragung der Studierenden wurde durch den Bereich Evaluation und Kapazität des Studiendekanats der MHH genehmigt. Die Auswertung wurde mit SPSS Version 25.0 durchgeführt. Die Analyse nutzte deskriptive, statistische Verfahren zur Berechnung der Häufigkeitsverteilung, Mittelwerte und Standardabweichung für alle geschlossenen Fragen. Die Antworten der offenen Fragen wurden vom Erstautor zusammengefasst und ausgewertet.

## Ergebnisse

**Figure 2:**
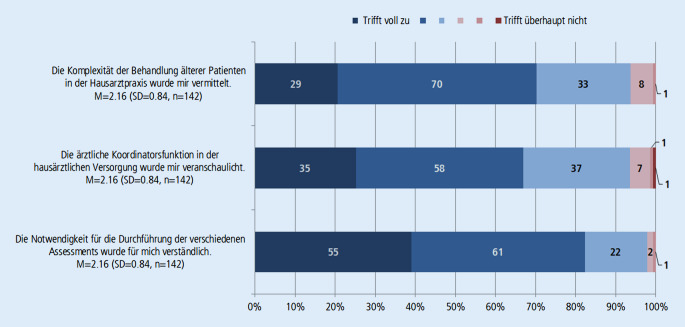

Von 295 befragten Studierenden haben 144 an der Evaluation teilgenommen. Der überwiegende Teil der Studierenden stimmt den Aussagen zu, dass der Lehrfilm die Komplexität der Behandlung älterer Menschen, die Koordinationsfunktion des Hausarztes und die Notwendigkeit der Durchführung von verschiedenen Assessments anschaulich vermittelt (Abb. 2). Unter den Befragten hatten nur 5 Studierende noch keinen Kontakt zu älteren Patient*innen. Der überwiegende Teil der Studierenden (82 %) war davon emotional berührt, Herrn K. dabei zuzusehen, wie er den Uhrentest macht. Von den Studierenden sagten 58 % aus, dass ihr Ersteindruck von Herrn K. nicht dem Ergebnis des Assessments entsprach (Abb. 4). Insgesamt bewerten die Studierenden den Lehrfilm auf einer 6‑stufigen Schulnotenskala durchschnittlich mit 1,95 (SD = 0,7, *n* = 139).

Von 77 Studierenden liegen Freitextkommentare vor.

### Rolle des GBa in der allgemeinmedizinischen Versorgung.

In 15 Formulierungen wird überwiegend positiv auf die Relevanz des geriatrischen Assessments Bezug genommen. Die Studierenden schreiben, dass der Film einen „gute[n] Eindruck von den Arztaufgaben“ [Stud.42] vermittelt, und einen „Überblick in die Methoden des Demenz-Screenings“ [Stud.117] gibt. Positiv hervorgehoben werden „das genaue Durchführen der Tests“ [Stud.99] und „die Arbeit des Hausarztes im privaten Umfeld zu sehen“ [Stud.91]. Bemerkt wird aber auch, dass die „besondere Rolle des Hausarztes eher im Hintergrund blieb“ [Stud.33].

### Beurteilung der Authentizität.

In den 108 Freitextkommentaren wird die realistische bzw. authentische Darstellung erwähnt. Dies zeigt sich in Formulierungen, dass es sich dabei um eine „realistische Durchführung des Assessments“ [Stud.33] und um „kein Schauspiel“ [Stud.125] handele, „dass es nicht ‚gestellt‘ war“ [Stud.10] oder „nicht gespielt“ [Stud.126] sei. Der Film „wirkt authentisch“ [Stud.57], es gäbe „keine gestellte Szene (wirkte zumindest nicht so)“ [Stud.49], da auch „keine Schauspielpatienten“ [Stud.52] zum Einsatz kämen. Gegenteilige Charakterisierungen des Films, als eher nicht realistisch, werden zumeist in Bezug auf Einzelaspekte formuliert: „recht lang, die Ratschläge bzgl. der Wohnraumgestaltung waren unrealistisch“ [Stud.59].

### Emotionale Wirkung.

Die emotionale Wirkung wird mit Aussagen zum Ausdruck gebracht, in denen ein für die Studierenden unerwarteter Verlauf des Assessments geäußert wird. Unter den positiven Bewertungsaspekten befinden sich 7 Freitextkommentare, in denen die Studierenden von einem anderen Ersteindruck des Patienten Herr K. und ihrer Überraschung hinsichtlich des Assessmentergebnisses sprechen. Berührend sei es, „zu sehen, wie viel die „Beziehung“, das Vertrauen zwischen Patienten und Arzt in dem Kontext des allgemeinmedizinischen Hausbesuchs bedeutet“ [Stud.48]. Der Film vermochte es, sich „… sehr gut in die häusliche Situation des Patienten hineinzuversetzen“ [Stud.94].

## Diskussion

Mit dem Einsatz und der Evaluation eines Lehrfilms zum GBa sollte untersucht werden, ob mit filmischen Mitteln ein authentischer Eindruck vermittelt werden kann, der von den Studierenden nicht nur als realistische Darstellung der Aufgaben der allgemeinmedizinischen Versorgung älterer Menschen wahrgenommen wird, sondern auch die emotionale Wahrnehmung der Situation erlaubt und so für die Probleme älterer Menschen sensibilisiert.

Der Hausbesuch bei geriatrischen Patientinnen und Patienten als Teil der curricularen Lehre [13, 21] bietet sicherlich den unmittelbarsten und authentischsten Einblick, um eine bessere Vorstellung der Lebensrealität Älterer zu erlangen, wie Weltermann et al. [22] fordern. Dieser direkte Kontakt ist gerade bei großen Kohorten aber nur schwierig zu realisieren. Alternativen, die sich auch bewährt haben, sind z. B. der Einsatz von Simulationspatienten, etwa in einem Lehrfilm, in dem die geriatrische Untersuchung mit Simulationspatienten nachgespielt wird [4], oder die Übernahme der Rolle des Patienten durch die Studierenden selbst [16], indem verschiedene Hilfsmittel genutzt werden, um altersbedingte Funktionseinschränkungen erlebbar zu machen. Ein authentischer Eindruck älterer Patienten kann aber nur bedingt durch Simulationen erreicht werden, wie Experimente von Bokken et al. zeigen [3].

Den Lehrfilm mit realen Patienten zu drehen, hatte daher sicherlich einen starken Einfluss auf die Wahrnehmung des Geschehens. Die Ergebnisse der Evaluation zeigen, dass ein Lehrfilm, der gezielt verschiedene filmische Mittel zur Erreichung von Authentizität einsetzt, einen realistischen Eindruck von der Komplexität und Notwendigkeit des GBa vermitteln kann (Abb. 2 und 3) und dabei eine emotionale Betrachtung der Situation für die Studierenden ermöglicht (Abb. 4).

**Figure 3:**
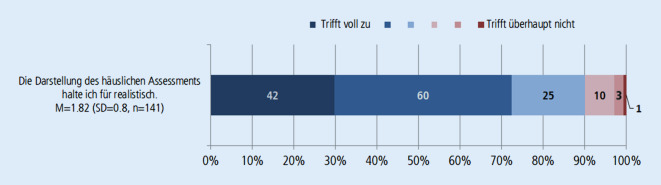

**Figure 4:**
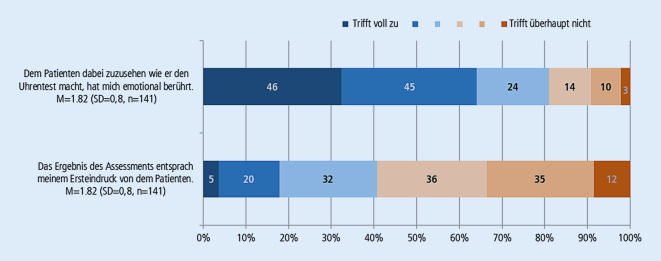

Dem filmischen Konzept der Authentizität entsprechend wurde das nichtvorhersehbare Assessmentergebnis mit der Darstellung des Scheiterns des Patienten an den an ihn gestellten Aufgaben ungeschnitten gezeigt. Im Sinne von Wuss kommt in den Aussagen der Studierenden zum Ausdruck, dass es sich bei dem „… Realitäts-Effekt nie um ein filmisches Strukturangebot per se handelt, sondern stets auch um das Resultat einer psychischen Aktivität des Zuschauers, etwa einer perzeptiven Anstrengung, die auf die notwendige Interaktion von Formgestalt und internem Modell des Zuschauers hinausläuft“ [25].

Trotz des realistischen Eindrucks muss berücksichtigt werden, dass die filmische Apparatur auf das Geschehen Einfluss nimmt. Dies zeigt sich darin, dass sich der Patient der Filmaufnahme bewusst ist. Dessen Reaktion auf die Kamera wird von Studierenden gerade als „realistische Darstellung“ betrachtet. Durch die Anwesenheit der Kamera sei er ein „aufgeregter Patient“ [Stud.144]. Diese Wahrnehmungen der Studierenden des realistischen filmischen Charakters belegen die individuellen, aber auch kulturhistorisch entwickelten und Veränderungen unterworfenen Sichtweisen auf Authentifizierungsstrategien [19]. Authentizität ist daher kein Qualitätsmerkmal des Mediums an sich [23].

Die Wirkungsweise der filmischen Darstellungsform lässt sich an den Empfindungen der teilnehmenden Studierenden bei der Wahrnehmung der maßgeblichen Assessments zur Erhebung der kognitiven Leistungsfähigkeit ermessen. Der Großteil der Studierenden war emotional berührt, Herrn K. dabei zuzusehen, wie er am Uhrentest scheitert. Auch wenn fast alle Studierenden bereits Kontakt mit älteren Patient*innen hatten, ist der auffälligste Wert der Befragung das Ergebnis zur Einschätzung der kognitiven Leistungsfähigkeit des Patienten. Die Mehrheit der Studierenden gab an, dass ihr Ersteindruck von Herrn K. nicht dem Assessmentergebnis entsprach. Die Beurteilung des Patienten ist demnach bei Fortschreiten des Films und der jeweiligen Assessmenttests zunehmend vom eingangs entstandenen Bild abgewichen. Zu Beginn zeigt der Film das Leben und Wohnen des Ehepaars, das trotz ihres hohen Lebensalters in geordneten Verhältnissen verläuft. Im gewohnten häuslichen Umfeld und im Zusammenspiel mit der Ehefrau findet Herr K. entscheidenden Rückhalt. Die Antworten der MAGIC-Testfragen offenbaren noch keine schwerwiegenden Probleme, zeigen lediglich weiteren Klärungsbedarf auf. Erst die Durchführung des Uhrentests von Herrn K. erhärtet den Verdacht einer kognitiven Einschränkung und wird zum zentralen Element des Assessments.

Um dem Stellenwert des Uhrentests gerecht zu werden, wird dieser im Lehrfilm in voller Länge abgebildet und nicht nur das Testergebnis angezeigt. Nicht selten werden auch Lehrfilme durch die Montage verdichtet. Im Unterschied dazu verfolgt der hier vorgestellte Film eine andere filmische Strategie und stellt eine direktere Wirklichkeitsrepräsentation dar. Der filmische Ansatz soll die tatsächliche Konstitution des Patienten nachvollziehbar machen. Insofern entspricht die Erzählzeit des Films exakt der erzählten Zeit, und die mühsamen Anstrengungen des Patienten bei der Bewältigung der an ihn gestellten Aufgabe werden erlebbar. Die Reaktionen der Studierenden auf diese Szene unterstreichen die Prozesshaftigkeit in der filmischen Rezeption. In der Filmtheorie schreibt Wuss dem Rezipienten in diesem Sinne einen wesentlichen Stellenwert bei der Bedeutungsproduktion von künstlerischen Werken zu [24]. Sein Modell fasst die Filmrezeption als einen mehrstufigen Lernprozess auf, der auch im Lehrsetting der medizinischen Ausbildung didaktisch genutzt werden kann.

### Limitationen

Trotz der Größe der befragten Kohorten weist die Studie im Hinblick auf die Zusammensetzung der Befragungsgruppe einige Limitationen auf. Alle Studierenden nahmen am Unterrichtsmodul im gleichen Lehrsetting teil, insofern gab es keine Vergleichsgruppe. Die befragte Kohorte war zwar groß, aber im Hinblick auf die Vorerfahrung mit älteren Patienten eine homogene Gruppe. Bei der Frage, ob die Studierenden bereits Kontakt zu älteren Patienten hatten, wurde zudem nicht weiter differenziert, in welcher Form und wie intensiv der Kontakt zu diesen war. Eine weitere Limitation besteht darin, dass für die Untersuchung nur subjektive Selbsteinschätzungen vorliegen, aber eine objektive Messung der Veränderung der Haltungen und des Wissenszuwachses nicht erfolgte.

### Ausblick

Unter Rahmenbedingungen, die eine weitestgehende Online-Lehre erfordern, erweisen sich die Produktion und der Einsatz von Lehrfilmen als äußerst nutzbringend. Allerdings muss das Lernszenario hierfür adaptiert und ein spezifisches E‑Learning-Konzept realisiert werden.

Um die Aussagekraft der Untersuchungsergebnisse zu erhöhen, wäre zu untersuchen, welchen Einfluss Emotionen, die im Lehrkontext durch den Einsatz von filmischen Realitätsausschnitten hervorgerufen werden, Einfluss auf die Entwicklung von Haltungen haben können. Eine vergleichende Untersuchung zwischen 2 studentischen Gruppen, bei der der ersten Gruppe der Film gezeigt wird und einer zweiten Kohorte nur die Ergebnisse des Assessments ohne die Bearbeitungsanstrengungen durch den Patienten, könnte Aufschluss darüber geben, welchen Einfluss die filmische Form auf die emotionale Wirkungsweise hat.

## Fazit für die Praxis

- Lehrfilme sind geeignet, um geriatrische und allgemeinmedizinische Themen sowie die ambulante Patientenversorgung in die medizinische Ausbildung zu transportieren. Sie machen die Versorgungsaufgaben von niedergelassenen Hausärzt*innen für Studierende erlebbar und sind ein geeignetes didaktisches Mittel, um zu aktivieren und individuelle Sichtweisen zu befördern.
- Schon bei der Konzeption eines Lehrfilms sollte die filmische Form berücksichtigt werden. Der Einsatz von realen Patient*innen kann die emotionale Wirkung von Lehrmedien verstärken, erfordert aber einen sensiblen und vertrauensvollen Umgang.
